# Radiocarbon Palaeolithic Europe database: A regularly updated dataset of the radiometric data regarding the Palaeolithic of Europe, Siberia included.

**DOI:** 10.1016/j.dib.2020.105793

**Published:** 2020-06-04

**Authors:** Pierre M. Vermeersch

**Affiliations:** a Department of Earth and Environmental Sciences, KU Leuven, Belgium; b Geo-Institute, Celestijnenlaan 200E, B-3001 Leuven, Belgium

**Keywords:** Palaeolithic, Radiometric dating, ^14^C dating, Europe, Quaternary, Prehistory, Archaeology

## Abstract

At the Berlin INQUA Congress (1995) a working group, European Late Pleistocene Isotopic Stages 2 & 3: Humans, Their Ecology & Cultural Adaptations, was established under the direction of J. Renault-Miskovsky (Institut de Paléontologie humaine, Paris). One of the objectives was building a database of the human occupation of Europe during this period. The database has been enlarged and now includes Lower, Middle and Upper Palaeolithic sites connecting them to their environmental conditions and the available chronometric dating. From version 14 on, only sites with chronometric data were included.

In this database we have collected the available radiometric data from literature and from other more restricted databases. We try to incorporate newly published chronometric dates, collected from all kind of available publications. Only dates older than 9500 uncalibrated BP, correlated with a "cultural" level obtained by scientific excavations of European (Asian Russian Federation included) Palaeolithic sites, have been included. The dates are complemented with information related to cultural remains, stratigraphic, sedimentologic and palaeontologic information within a Microsoft Access database. For colleagues mainly interested in a list of all chronometric dates an Microsoft Excel list (with no details) is available (Tab. 1). A file, containing all sites with known coordinates, that can be opened for immediate use in Google Earth is available as a *.kmz file. It will give the possibility to introduce (by file open) in Google Earth the whole site list in "My Places".

The database, version 27 (first version was available in 2002), contains now 13,202 site forms, (most of them with their geographical coordinates), comprising 17,022 radiometric data: Conv. ^14^C and AMS ^14^C (13,144 items), TL (678 items), OSL (1050 items), ESR, Th/U and AAR (2150 items) from the Lower, Middle and Upper Palaeolithic. All ^14^C dates are conventional dates BP. This improved version 27 replaces the older version 26.

Specifications Table

**Table:** 

Subject	Archaeology
Specific subject area	All chronometric data from Palaeolithic sites of Europe according to their archaeologic and stratigraphic level.
Type of data	Microsoft Access file
How data were acquired	In this database we have collected the available radiometric data from literature and from other more restricted databases. We try to incorporate newly published chronometric dates collected from all kind of available publications.
Data format	Raw The database uses Microsoft Access©. The database is available http://www.ees.kuleuven.be/geography/projects/14c-palaeolithic/index.html
Parameters for data collection	• Included are all radiometric data that can be correlated with the presence of Palaeolithic humans over Europe, Asian Russia included. The data are thus mainly available through the scientific excavations of Palaeolithic sites and can be related to a stratigraphy and eventually to a prehistoric “culture”. • The information collection is organised in following items: Geographical Information; Cultural Information; Stratigraphical information; Chronology with 1) Conventional ^14^C age in BP, 2) AMS age in BP, 3) TL age, 4) OSL age, 5) Other age (ESR, U-Series); Postdepositional Activities; Local Climate; Environment Condition; Bibliographic Reference. • The basic item for a single form of the database is a layer or a level from a specific site. • The different chronometric dates from a specific layer are grouped into a single form. • When a site is characterized by an extensive stratigraphic profile with many dates, we only take over those dates that relate to a prehistoric occupation level. • Only dates older than 9500 uncalibrated BP are included in the database. • If our source has a map of the site(s), we use Google Earth for a more precise location of the site and for the determination of longitude and latitude. When no details are available, the coordinates of the center of the village where the site is situated, are used. • When different cultural attributions are given in the literature, all of them will be given. • Unreliable results can be indicated (In the remarks a reason can be given). The reason for considering a date as unreliable is not our evaluation but that of one of the references cited. • Each radiometric date is given as such. It is not our purpose to evaluate the date. A poor dating procedure (e.g. too small sample, or incomplete treatment of the sample) can be mentioned. • When available, the sample treatment is given.
Description of data collection	The collection of the data is accomplished by the continuous checking of newly published articles in 400 international and regional scientific journals and in collections or books dealing with a particular period or a specific Paleolithic site. The list of journals inspected is available as "inspected-journals.xls" at http://www.ees.kuleuven.be/geography/projects/14c-palaeolithic/index.html. The radiometric dates mentioned therein are taken over in the database. Since the database has become known, colleagues have also made available newly acquired radiometric dating. When collecting the dates, care is taken to ensure that the dates are the result of careful sampling and of a standardized dating method in the various laboratories. The dates are included in the database as delivered by the laboratories. A further processing of the data, such as calibration, is not included. The data already present in the database are continuously updated and checked. After all, it turns out that specific published data sometimes differ from author to author. If this is the case, the authors are contacted to find out which of the given data is correct. The database is intended to be continuously supplemented and improved. As a result, the current data is already version 26, while the first version was created in 2002. The intention is to continue this process.
Data source location	KULeuven, Leuven, Country: Belgium
Data accessibility	Repository name: KULeuven (is not really a “Repository”)Data identification number: [provide number]Direct URL to data: http://www.ees.kuleuven.be/geography/projects/14c-palaeolithic/index.htmlIt is also available at Mendeley Data Contributors:Pierre VermeerschDate:2020–03–16Radiocarbon Palaeolithic Europe Database v26 jan 2020.mdb… Paleolithic Period… Radiocarbon Palaeolithic Europe Database v26 jan 2020 extract.xlsx… Rdiocarbon Palaeolithic Europe Database v26 jan. 2020docx.docx… Radiocarbon DatingData Types: • Software/Code • Geospatial Data • Tabular Data • Dataset • Document Our dates (older version) are included in the “Canadian Archaeological Radiocarbon Database (CARD 2.0)” http://www.canadianarchaeology.ca/.
Related research article	Vermeersch Pierre M., Josette Renault-Miskovky. 1999. *European late Pleistocene, isotope stages 2 and 3 : Humans, their ecology & cultural adaptations, inqua Congress in Durban south Africa, 3–11 Augst 1999*, international Union for quaternaty research/Union internationale pour l’étude du quaternaire, Commitee on human evolution & palioecology, Liège, ERAUL 9O, 242 p. Vermeersch P.M. 2005. European population changes during Marine Isotope Stages 2 and 3. *Quaternary International* 137: 77–85*.*

Value of the Data

• They bring together what is spread over a large often not easily to locate literature. The database is larger (more information) than most databases provided by the dating laboratory and includes succinct but important archeological data fitting within the chronological data.
• Other researchers working on geographical spread, cultural development of the Palaeolithic in Europe have an easy access to available dates form sites, individualised by cultural level.
• As the database is updated on a regular basis, it is a good easy to access and increasingly reliable basis for finding the elements of the dating of the Palaeolithic in Europe.
• The value of the database is best explained by the use it had already in numerous scientific contributions from high ranking journals [1], [2], [3], [4], [5], [6], [7], [8].

## Data description

**Fig. 1:**
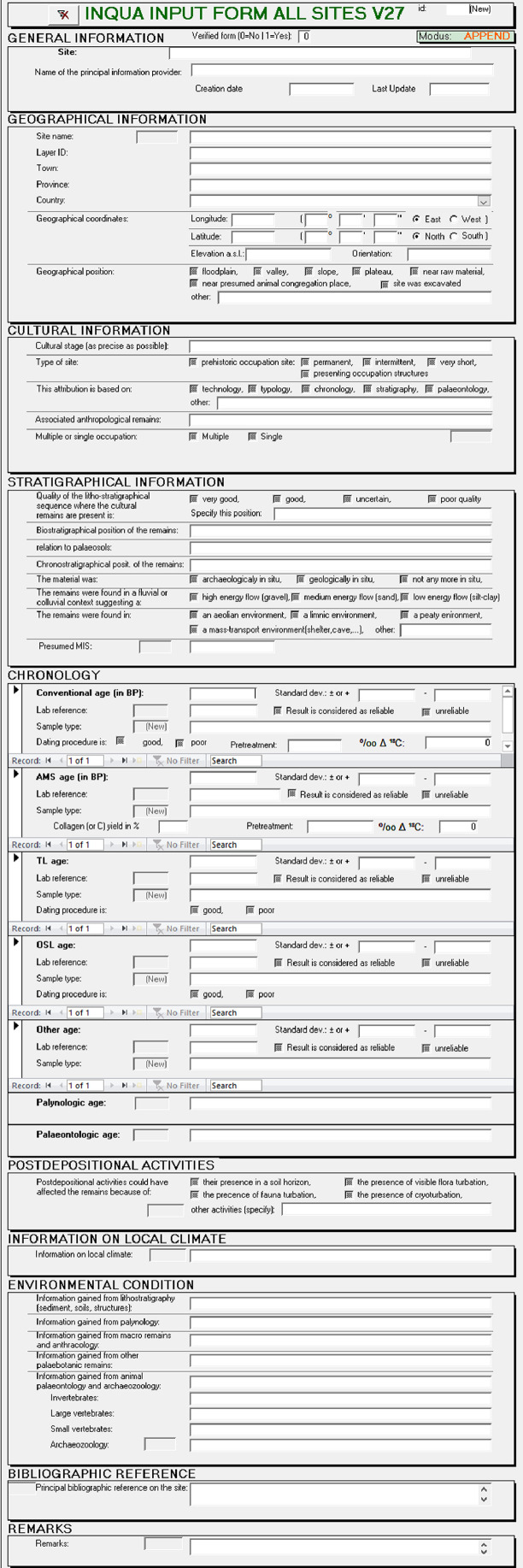
The lay out of the input form A high number of queries (Fig. 2) is available facilitating the selection of specific European countries or cultures such as Acheulean, Middle Palaeolithic, Aurignacian, Gravettian etc.

• The content of the data can best been described by a copy of the input form (Fig. 1) in a Microsoft Access file, which contains following items Geographical Information; Cultural Information; Stratigraphical information; Chronology with 1) Conventional ^14^C age in BP, 2) AMS age in BP, 3) TL age, 4) OSL age, 5) Other age (ESR, U-Series); Postdepositional Activities; Local Climate; Environmental Condition; Bibliographic Reference.

**Fig. 2:**
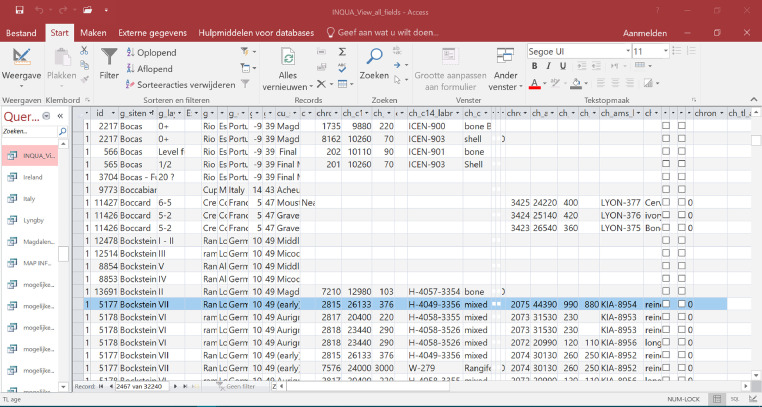
A rapid overview of the available data by a query A *kmz file (Fig. 3) gives a rapid overview of the sites distribution with their cultural attribution in Google Earth.

**Fig. 3:**
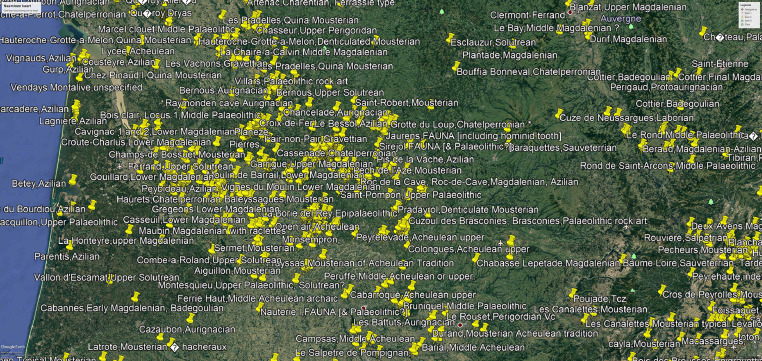
Google Earth map from the region of Southwest France with the position of the sites.

## Experimental design, materials, and methods

As stated above the database is entirely based on the analysis of scientific literature which is accessible through University libraries, internet, contact with colleagues etc. The information is scanned by reading the contributions.

## Declaration of Competing Interest

No competing interests

No financial support is available.

The author declares that he has no known competing financial interests or personal relationships which have, or could be perceived to have, influenced the work reported in this article.

## Appendix Supplementary materials

Image, application 1

Image, application 2
